# The effects of an integrated mindfulness-based tai chi chuan programme on sleep disturbance among community-dwelling elderly people: protocol for a randomized controlled trial

**DOI:** 10.1186/s13063-022-06737-4

**Published:** 2022-09-24

**Authors:** Sunny Ho-Wan Chan, Siu-Man Ng, Chong-Ho Yu, Ching-Man Chan, Shu-Mei Wang, Wai-Chi Chan

**Affiliations:** 1 School of Health and Social Wellbeing, University of the West of England, Bristol, UK; 2 Department of Social Work and Social Administration, The University of Hong Kong, Pok Fu Lam, Hong Kong; 3 Department of Psychology, The Azusa Pacific University, Azusa, USA; 4 Division of Elderly Service, Yang Memorial Methodist Social Service, Yau Ma Tei, Hong Kong; 5 Department of Rehabilitation Sciences, The Hong Kong Polytechnic University, 11 Yuk Choi Rd, Hung Hom, Kowloon, Hong Kong; 6 Department of Psychiatry, The University of Hong Kong, Pok Fu Lam, Hong Kong

**Keywords:** Mindfulness-based tai chi chuan, Community-dwelling elderly, Mind-body interventions, Sleep disturbance, Randomized controlled trial

## Abstract

**Background:**

Many elderly individuals who experience sleep disturbances would consider complementary and alternative medicine as an alternative therapeutic option in light of the limitations of traditional treatments. Mindfulness-based interventions (MBIs) and Tai Chi Chuan (TCC) are two alternative forms of complementary and alternative medicine. They both share the common feature of a focus on breathing but represent distinct approaches with different mechanisms and philosophical orientations. The trial described in this protocol aims to evaluate the effects of an integrated form of mindfulness-based Tai Chi Chuan (MBTCC) programme and the underlying mechanisms of the beneficial effects over a 12-month follow-up.

**Methods:**

The planned study is a four-armed randomized controlled trial with repeated measures. A total of 256 community-dwelling older adults with sleep problems will be recruited and randomized into four groups: (1) an MBTCC group, (2) an MBI group, (3) a TCC group, and (4) a sleep hygiene education (SHE) control group. The outcome measures in terms of insomnia severity, interoception, sleep-wake pattern, health status, rumination, and hyperarousal level will be collected at four time points: at baseline (T1), after the 8-week intervention (T2), 6 months after the intervention (T3), and 1 year after the intervention (T4). In addition, qualitative evaluation through focus group interviews will be conducted at the end of the 12-month assessment period (T4).

**Discussion:**

This trial will illuminate the synergetic effect of combining both MBIs and TCC on optimizing improvements in sleep disturbance. The findings from this study can provide empirical support for this integrated treatment, which provides an alternative for healthcare professionals in elderly service to select appropriate practices to treat elderly people with sleep disturbance. It can further help to lessen the growing public health burden of sleep disturbances among the elderly living in the community.

**Trial registration:**

ClinicalTrials.gov. NCT05396092. Published on 24 May 2022

## Background

Sleep problems are common during the ageing process and can result in fatigue, mood disturbances, problems with interpersonal relationships, and even a reduced quality of life [1]. Sleep problems are often characterized by subjective complaints about dissatisfaction with sleep quality or duration, difficulty falling asleep at bedtime, waking up in the middle of the night or too early in the morning, and non-restorative sleep [2]. Evidence indicates that sleep changes with increasing age such as more sleep fragmentation or earlier awakening may be due to the chronobiological changes taking place in the physiological systems that accompany the ageing process [3]. If left untreated, it has been reported that insomnia can seriously impact depression and anxiety among the elderly [4]. A territory-wide survey indicated that the overall prevalence rate of poor sleep quality for community-dwelling Chinese older adults was 42%. In addition, the prevalence rate was shown to increase with age, from 32% in those aged 60–69 years to 53% in those aged ≥ 80 years [5]. Due to varied operational definitions of sleep problems (e.g. insomnia as a symptom versus a diagnosis), the term “sleep disturbance” [6] is used in this proposal to describe both the clinical and subclinical experiences of sleep problems.

Pharmaceutical treatment is the most common method used to improve sleep quality. However, it has severe limitations, due to the presence of potential drug dependence and concomitant side effects [7]. Emerging psychological and behavioural treatments for sleep disturbances have thus been growing in recent decades. A meta-analysis indicated that cognitive behavioural therapy is an effective non-pharmacological intervention that can be used to treat chronic insomnia, with moderate to large effect sizes on the outcomes [8]. However, not all patients respond to these interventions [2]. Moreover, previous research has shown that only 26 to 43% of older adults achieve full remission from insomnia through cognitive behavioural therapy [9, 10]. Thus, there remains a critical need to develop and evaluate the efficacy of alternative treatment options to address the continued substantial public health burden of sleep disturbances.

Due to the limitations of traditional treatments, many elderly individuals who experience sleep disturbances are willing to consider using complementary and alternative medicine (CAM) as an alternative therapeutic option, including natural herbal products, acupuncture, or mind-body interventions, for example. A national health interview survey revealed that approximately 1.6 million American adults use CAM therapies to treat insomnia or problems with sleep [11]. A local community-based study showed that a high proportion of Hong Kong Chinese middle-aged adults (35%) would seek help from CAM for their sleep problems [12]. As sleep problems become increasingly severe with age, there may be a burgeoning trend of elderly individuals seeking alternative treatments for their sleep problems. Among different CAM therapies, the biologically based and mind-body domains are by far the most commonly used [11].

Mindfulness-based interventions (MBIs), as a kind of CAM mind-body treatment with a focus on changing cognitive processes (such as “acceptance”), have become increasingly popular and are now used to treat a number of health problems, including sleep disturbances. MBIs have received a great deal of attention in the fields of neuroscience and psychology over the past few decades. Mindfulness (Pali: *sati*) originates in Buddhism, signifying the seventh factor in the noble eightfold path. As such, mindfulness can be defined as the process of deliberately cultivating non-judgemental moment-to-moment awareness and experiences, through observing one’s own mind in a detached manner [13]. Tai Chi Chuan (TCC) can be viewed as another alternative CAM mind-body intervention, with emphasis placed on the body or movement. TCC, a form of traditional Chinese martial arts, originates from Taoism and concerns the essence of harmony. It is widely available and accessible to older adults in community settings. The emphasis of TCC is on movement-based practice. It involves slow-paced physical movements that are practised concomitantly with an introspective focus and an awareness of breathing, as well as the natural force or energy in the body. TCC emphasizes the integration of flowing movements, dynamic and static postural training, and focused mental attention [14].

**Fig. 1:**
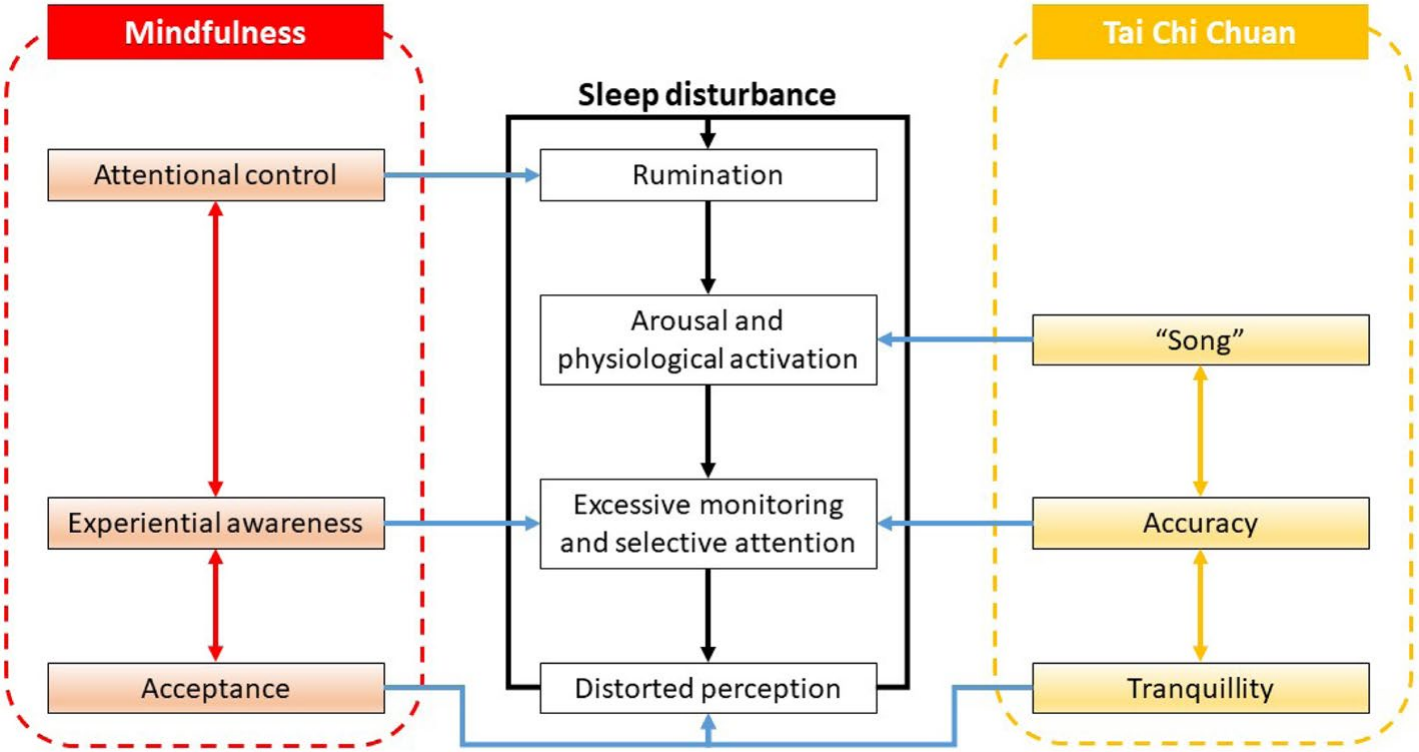
Theoretical framework about the mechanisms of MBI and TCC in relation to sleep disturbances. MBI, mindfulness-based intervention; TCC, Tai Chi Chuan

An aetiological model of sleep disturbance [6] can lay the groundwork for a better understanding of the mechanisms of MBIs and TCC in relation to sleep disturbances (Fig. 1). Basically, sleep disturbances are instigated by sequential cognitive, physiological, and behavioural processes, including (1) excessive rumination; (2) pre-sleep arousal, leading to distress and physiological activation; (3) the excessive monitoring of and selective attention to internal bodily sensations and/or external sleep cues; and (4) distorted perceptions about sleep impairment. Misperceptions regarding sleep deficits thus lead to excessive negative cognition and result in a vicious cycle of sleep disturbances.

The three core principles of mindfulness—experiential awareness, attentional control, and acceptance—can target different vulnerabilities associated with sleep disturbances. More specifically, the cultivation of experiential awareness should promote awareness of a range of experiences, including internal and external stimuli, which can reduce excessive monitoring and selective attention. Besides, attentional control, achieved by focusing attention on the breath (sustained attention) and redirecting attention to the anchor whenever one encounters wandering thoughts (attention inhibition), should enable individuals to disengage from negative thoughts. In addition, focusing on the present moment should help individuals let go of excessive rumination and monitoring. Finally, acceptance can be learned by non-judgementally viewing the experience of observing one’s own thoughts and feelings as passing events, rather than as facts. Acceptance should also lead to equanimity, regulating individuals’ deformed perceptions of sleep disturbances [6].

The three interdependent ideologies of TCC (accuracy, “song”, and tranquillity) [14] can also impact various processes associated with sleep disturbances. Specifically, a great deal of emphasis has been placed on the accurate performance of TCC postures, along with the internal qualities of the activity. These postures can allow Qi (energy) to pass smoothly through the correct alignment. The high level of concentration on bodily posture and movement should help reduce excessive monitoring and enhance focused attention. Another core principle strived for in TCC training is “song”, which can be interpreted as a quality that is experienced in a light, free, open, and effortless manner; at the same time, it is stable, powerful, and well-rooted. The achievement of “song” through the employment of a relaxed and apparently effortless posture should attenuate arousal-related responsiveness and related physiological responses. Paying attention to the accuracy and “song” of posture and movement leads to a state of tranquillity. Ultimately, TCC should lead to a state of “emptiness” and “non-doing”, which should help settle the unease caused by sleep disturbances.

The benefits of both MBIs and TCC are reflected in previous research findings. A recently conducted meta-analysis indicates that MBIs show promising effects in regard to the improvement of sleep disturbances [15]. Nevertheless, evidence supports the way in which MBIs are associated with improvements in self-reported outcome measures only [15]. In addition, a recent local study has investigated the effects of mindfulness-based cognitive therapy on insomnia, as compared with sleep psycho-education with an exercise control condition [16]. The investigators found that mindfulness-based cognitive therapy demonstrated short-term benefits for sleep quality only. It is possible that MBIs targeting only cognitive and behavioural vulnerabilities associated with sleep disturbances may therefore produce only limited benefits. Additional focus on physiological and arousal levels may be needed to boost the long-term effects of MBIs. TCC has been shown to emphasize individuals’ control over physical health function and arousal-related responsiveness. Research indicates that TCC provides physiological evidence of its benefits on the autonomic nervous system [17]. A systematic review also found significant benefits of TCC on sleep problems among older adults. However, this evidence is still inconclusive because of the varying quality of these studies and the insufficient fidelity of the intervention implementation to confirm the results [18].

MBIs and TCC represent two distinct forms of mind-body interventions, but they can have a supplementary effect on each other and with a synergetic contribution to ameliorating sleep disturbances. When combining the strength of these two interventions, this integrated intervention may yield a greater treatment effect than using either treatment alone. Therefore, we expect that the integrated mindfulness-based Tai Chi Chuan programme should be more effective and feasible as applied locally.

## Methods

### Study aims

The following are the study aims:

- To evaluate the effects of an integrated mindfulness-based Tai Chi Chuan (MBTCC) programme on the improvement of insomnia and other secondary outcomes over a 12-month follow-up period, by comparing it to an MBI-alone group, a TCC-alone group, and a sleep hygiene education (SHE) control group among community-dwelling elderly individuals experiencing sleep disturbances.
- To explore the mediating roles of rumination and psychological function as mechanisms underlying the beneficial effects of the MBI-alone group on insomnia severity.
- To explore the mediating roles of arousal level and physical function as mechanisms underlying the promising effects of the TCC-alone group on insomnia severity.
- To explore the mediating roles of all the proposed mediators underlying the favourable effects of the MBTCC group on insomnia severity.
- To identify and understand the therapeutic ingredients and conditions that contribute to the beneficial effects of the intervention through a qualitative evaluation.

### Hypotheses

- Hypothesis 1. Participants in the MBTCC group will report significantly greater improvements at 8-week (T2), 6-month (T3), and 12-month (T4) follow-ups in regard to measures of the primary outcome of insomnia severity, compared with participants in the MBI-alone group, the TCC-alone group, and the SHE control group.
- Hypothesis 2. Participants in the MBI-alone group will experience significantly greater improvements than those in the SHE control group in the primary outcomes across time, in which the beneficial effects on insomnia severity will be mediated by rumination and psychological function.
- Hypothesis 3. Participants in the TCC-alone group will experience significantly greater improvements than those in the SHE control group in the primary outcomes across time, in which the promising effects on insomnia severity will be mediated by the arousal level and physical function.
- Hypothesis 4. The favourable effects of the MBTCC group on insomnia severity will be mediated by all the proposed mediators.

### Study design

**Fig. 2:**
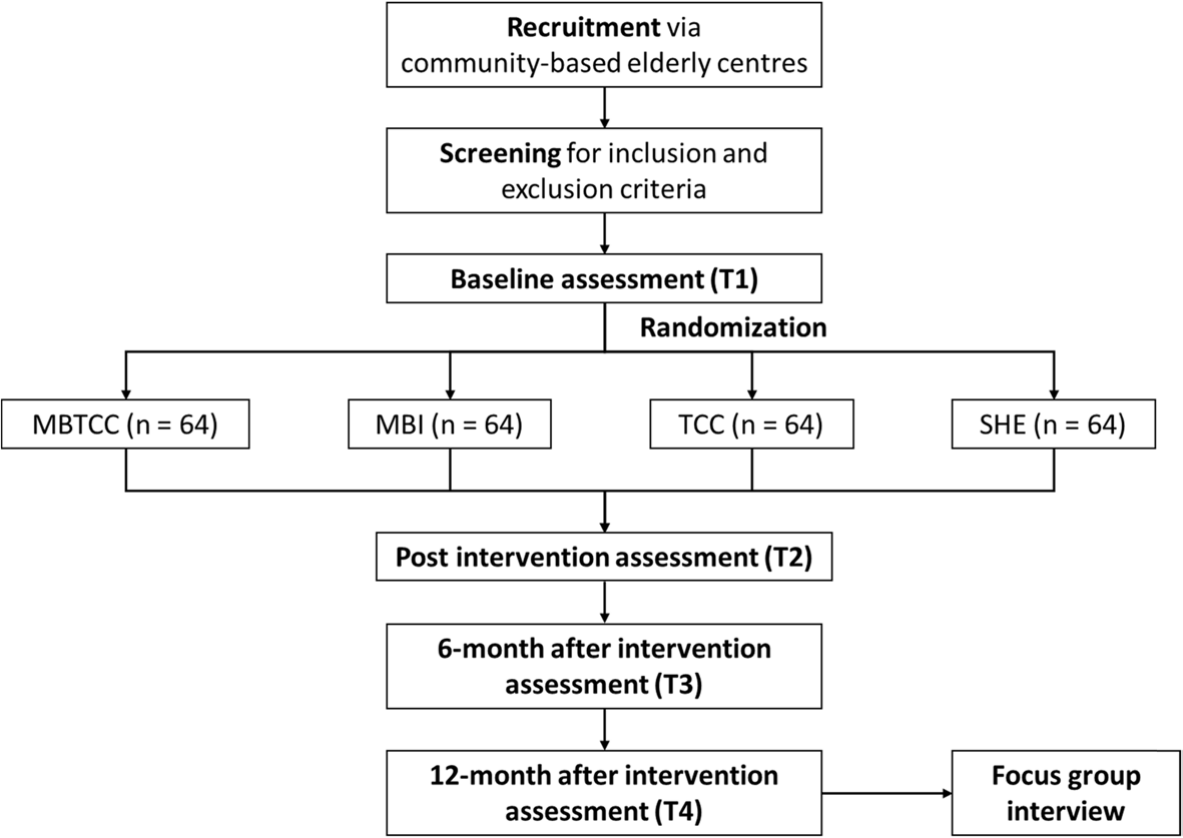
Flow chart of the study. MBTCC, mindfulness-based Tai Chi Chuan; MBI, mindfulness-based intervention; TCC, Tai Chi Chuan; SHE, sleep hygiene education

Because both quantitative and qualitative data will be collected, this study has a sequential explanatory design. This is a superiority trial, with equal allocation to the intervention and control groups. Specifically, qualitative data will be collected after the quantitative phase is finished, and qualitative data with rich contexts will be used to illuminate the quantitative findings. The current quantitative study is a randomized controlled trial with four study arms: (1) a MBTCC group, (2) a MBI group, (3) a TCC group, and (4) a SHE control group. The outcome measures will be collected at four time points: at baseline (T1), after the 8-week intervention (T2), 6 months after the intervention (T3), and 1 year after the intervention (T4). In addition, qualitative evaluation through focus group interviews will be conducted at the end of the 12-month assessment period (T4). The study flowchart is displayed in Fig. 2.

### Participants

Participants will be recruited from community-based elderly centres in Hong Kong. Specifically, three major elderly centres as managed under three non-government organizations (i.e. Yang Memorial Methodist Social Service, Evangelical Lutheran Church Social Service, and Hong Kong Sheng Kung Hui Welfare Council) will be approached. These three centres are in Kowloon, New Territories, and Hong Kong Island, respectively. Social workers from corresponding centres will be approached with an introduction to the research project. They will help recruit suitable participants in their respective centres. Initial screenings will be performed by the social workers according to our inclusion and exclusion criteria. The inclusion criteria are as follows: (1) aged 60 years or above, (2) classified as experiencing sleep disturbances (indication of poor sleep quality through a score > 5 in the Pittsburgh Sleep Quality Index [19]), (3) primary education level or above and able to communicate in Cantonese, (4) able to give informed consent, and (5) no previous experience with meditation or other mind-body techniques (e.g. tai chi or yoga). Elderly people with comorbid diagnoses of schizophrenia, schizoaffective disorder, substance misuse, organic brain syndrome, or intellectual disabilities will be excluded. Eligible participants will be further interviewed by the principal investigators or a research assistant in order to further explain to them the research project, to seek informed consent, and to complete the baseline assessments. After the initial baseline assessment, participants will be randomly assigned to one of the study arms using a list of computer-generated random numbers. The generation of random numbers and their assignment will be performed by a statistician who is unaware of the study aims. Another research assistant, who will assist with the outcome assessment and data analysis, will be kept unaware of the group allocation results. In order to promote participant retention and complete follow-up, the participants will be given cash coupons to thank them after completing the corresponding assessment at each time point. Besides, monthly telephone follow-ups will be conducted by a research assistant to remind the participants to do the home practice.

### Sample size

A four-armed randomized controlled pilot trial was recently conducted by the authors. A total of 76 community-dwelling older adults with symptoms of sleep disturbance took part in the pilot trial, with an attrition rate of 30%. They were randomly assigned to four different groups, including a MBI group, a TCC group, a MBTCC group, and a SHE group. The results of this pilot study are summarized in Table 1. Although the pilot study yielded moderate to large effect sizes (between .42 and .78), this variation implies that the effect sizes might be sample-dependent or context-dependent. In the current study, a larger sample size is therefore considered to be more desirable because it could pave the way for the identification of a small and hidden effect. Hence, in this power analysis, the effect size is set to .25 and the alpha level is .05. It is suggested that, to obtain the power level of .86 for the main effect and .97 for the interaction effect, at least 180 total participants are needed. Given that this study is longitudinal in nature and attrition (assuming the rate is 30%) is likely, an additional 76 participants will be recruited, resulting in a total of 256 participants (64 per arm).

**Table 1:** Comparison between four study arms in a pilot study

Variable	MBI group (*n* = 22)		TCC group (*n* = 23)		MBTCC group (*n* = 10)		SHE group (*n* = 21)		Effect size (MBI vs SHE), Cohen’s *d*	Effect size (TCC vs SHE), Cohen’s *d*	Effect size (MBTCC vs SHE), Cohen’s *d*	Effect Size (MBI vs TCC), Cohen’s *d*
	Pre, mean (SD)	Post, mean (SD)	Pre, mean (SD)	Post, mean (SD)	Pre, mean (SD)	Post, mean (SD)	Pre, mean (SD)	Post, mean (SD)				
ISI	17.27 (3.69)	15.64 (3.44)	17.22 (3.59)	15.70 (3.21)	17.10 (3.35)	14.40 (3.10)	16.95 (3.28)	16.90 (3.55)	− 0.44	− 0.42	− 0.78	− 0.03
SF12 (Phy)	11.05 (2.75)	11.55 (2.06)	11.48 (2.88)	12.91 (2.91)	11.90 (2.60)	13.90 (2.23)	12.76 (3.39)	12.52 (3.27)	0.24	0.52	0.69	− 0.32
SF12 (Men)	16.18 (3.66)	18.05 (3.75)	16.09 (3.40)	16.96 (3.05)	17.40 (3.20)	19.50 (3.21)	17.67 (3.90)	17.95 (3.50)	0.41	0.16	0.48	0.28
RRQ	3.06 (.79)	2.61 (.77)	2.89 (.88)	2.79 (.78)	3.23 (1.03)	2.68 (.84)	3.13 (.83)	3.10 (.66)	− 0.51	− 0.08	− 0.57	− 0.41
HS	62.23 (6.75)	57.59 (6.29)	61.39 (6.89)	53.52 (6.90)	64.00 (5.94)	54.70 (6.93)	63.29 (5.88)	61.48 (6.77)	− 0.44	− 0.93	− 1.24	0.47

*MBI* mindfulness-based intervention, *TCC* Tai Chi Chuan, *MBTCC* mindfulness-based Tai Chi Chuan, *SHE* sleep hygiene education, *ISI* Insomnia Severity Index, *SF12 (Phy)* Chinese (HK) Short Form 12 – Physical health, *SF12 (Men)* Chinese (HK) Short Form 12 – Mental health, *RRQ* Rumination-Reflection Questionnaire, *HS* hyperarousal scale

### Interventions

#### The MBTCC group

MBTCC will integrate components from both the MBI and TCC groups, as illustrated in the following. The entire intervention consists of eight weekly sessions of 2.5 to 3 h each. Details are shown in Table 2.

**Table 2:** Overview of different interventions

Session	MBI	TCC	MBTCC	SHE
1	● Awareness and automatic pilot ● Mindful eating ● Body and breath meditation	● Learning standing pose ● First posture: beginning Tai Chi ● Second posture: gasp sparrow’s tail	● Approaching mindfulness and TCC ● Mindful standing ● Awareness of breathing ● Practice of the first two postures of TCC	● General introduction of sleep disturbances and sleep hygiene ● Diaphragmatic breathing
2	● Keeping the body in mind ● Body scan ● Sitting meditation	● Standing pose ● First two postures ● Third posture: single whip	● Combining the mind and body together ● Mindful sitting ● Practice of the first three postures of TCC	● Understanding of sleep ● Practice of diaphragmatic breathing
3	● Gathering the scattered mind ● Mindful stretching ● Mindful walking	● Learning twisting pose ● Standing pose ● First three postures ● Fourth posture: lift hand upward	● Joining mindfulness and TCC ● Mindful twisting ● Practice of the first four postures of TCC	● Promoting habits and sleep environment ● Systematic muscular relaxation
4	● Expanding awareness ● Sounds and thoughts meditation ● 3-min breathing space	● Twisting pose ● Standing pose ● First four postures ● Fifth posture: white crane spreads wings	● Awareness expansion ● Mindful walking ● Practice of the first five postures of TCC	● Strengthening bed and bedroom as sleep stimulus ● Practice of systematic muscular relaxation
5	● Allowing/letting be ● Meditation in exploring difficulty ● Breathing space meditation	● Learning walking pose ● Standing pose ● First five postures ● Sixth posture: step forward, deflect, parry, punch	● Living with difficulties ● Learning about accuracy in TCC ● Practice of the first six postures of TCC	● Restricting time in bed ● Imagery rehearsal training
6	● Befriending meditation ● Loving-kindness and compassion ● Cultivating friendship towards oneself	● Walking pose ● Twisting pose ● First six postures ● Seventh posture: like sealing, as if closing	● Self-compassion ● Learning about “song” in TCC ● Practice of the first seven postures of TCC	● Diet relating to sleep ● Practice of imagery rehearsal training
7	● How can I best take care of myself? ● Decision-making in nourishing or depleting	● Standing, walking, or twisting pose ● First seven postures ● Eighth posture: cross hands	● Self-care ● Learning about acceptance in MBI ● Learning about tranquillity in TCC ● Practice of the first eight postures of TCC	● Day time exercises ● Worry containment and management ● Practice of different relaxation methods
8	● Building a precious life ● Weaving mindfulness into daily life	● Standing, walking, or twisting pose ● First eight postures ● Last posture: conclude Tai Chi	● Extending the practice of MBTCC to daily life ● Practice of the whole sets of TCC	● Building up a good and realistic sleep-wake habit ● Relapse management

*MBI* mindfulness-based intervention, *TCC* Tai Chi Chuan, *MBTCC* mindfulness-based Tai Chi Chuan, *SHE* sleep hygiene education

#### The MBI group

The MBI group is mainly based on the basic theory and research of the foundational mindfulness-based stress reduction programme [13]. Within this group, participants will cultivate the learning of mindfulness through various kinds of mindfulness activities, such as body scans, sitting meditation, mindful movements, mindful walking, and mindful eating. The intervention consists of eight weekly sessions of two to two-and-a-half hours each. Modifications to the content were carried out, to make it more suitable for elderly people with sleep disturbances. Information sheets about the MBI will be given to the participants. In order to facilitate practice through homework, each participant will be provided with an audio clip, which will be uploaded to their smartphones. All participants will be advised to practise mindfulness meditation at home on a daily basis, using the audio clip, and record it in a logbook.

#### The TCC group

TCC is mainly based on a nine-form Yang-style form of TCC, which is a brief and modified version of 64-form Yang-style TCC. It can be easily learned and practised without restrictions on time and space. Within the group, participants will be guided through learning the nine sequential forms of TCC movements. In addition, they will learn TCC post-standing, TCC walking, and TCC twisting. The essence of accuracy, “song”, and tranquillity will also be cultivated throughout the sessions. The entire intervention consists of eight weekly sessions of two to two-and-a-half hours each. Information sheets about the principles and skillsets of each form of TCC will be provided to each participant. Video clips will be provided and uploaded to participants’ smartphones to enable home practice. They will be advised to participate in daily home practice and keep a record of their daily practice.

#### The SHE control group

SHE is based on principles used in stimulus control and sleep hygiene education, which has previously been used as the control group in sleep-related research [20]. General guidelines include (a) go to bed only when sleepy at night; (b) get out of bed and leave the bedroom when unable to fall asleep or go back to sleep within 15–20 min and return to bed only when sleepy again; (c) do not use the bed/bedroom for activities (e.g. watching television, listening to the radio, eating, or reading in bed) other than sleep and sexual activities; (d) get up at the same time every morning; (e) do not nap during the day; and (f) do not compensate for lost sleep during holidays. Other information on the effects of external stimuli, such as environment and relevant relaxation techniques, will also be given and taught to participants. The entire intervention consists of eight weekly sessions of two to two-and-a-half hours each. They will be advised to follow the taught guidelines on a daily basis and keep a record.

#### Intervention and assessment fidelity

Each group will be taught by two qualified instructors and each session will be conducted in a similar amount of contact time. Each programme will consist of eight weekly sessions of 2 to 3 h each (16 to 24 h in total). There will be eight to 12 participants in each group. To ensure the fidelity of the intervention, qualified therapists with basic professional training in the teaching of mindfulness-based interventions, in addition to at least 2 years of experience conducting mindfulness-based programmes, will be invited to teach the MBTCC and MBI groups. Experienced Tai Chi masters will be invited to implement the MBTCC and TCC groups. The invited Tai Chi masters should have at least 10 years of experience of practice and teaching TCC. Experienced occupational therapists with at least 10 years of experience conducting mental health psychoeducation programmes will be invited to implement the SHE control group. The fidelity of each intervention programme will be ensured and monitored by a random review of two out of eight sessions. Based on the programme content of the respective intervention programmes, fidelity checks will be independently conducted by an experienced mindfulness instructor, an experienced Tai Chi master, or an experienced occupational therapist. For the qualitative part of the research project, the research personnel will receive half a day of training to learn the corresponding interview questions and the process for conducting the focus groups. In addition, regarding the qualitative analysis, another team of independent raters will audiotape, transcribe, and review the contents of the qualitative interviews to identify corresponding themes.

### Measurements

The psychometric attributes of all the measurement tools used in this project, such as reliability and validity, are psychometrically sound. For those assessments without a Chinese version, translation and cultural adaptation will be carried out. Outcome assessments will take place at T1 to T4 (i.e. T1, baseline; T2, after the 8-week intervention; T3, 6 months after intervention; T4, 12 months after intervention). All participants will be assessed in terms of the following aspects.

#### Screening measure

Sleep quality will be assessed by the Chinese version of the Pittsburgh Sleep Quality Index [19]. The index consists of a 19-item questionnaire evaluating sleep quality and disturbances over the past month. The first four items are open questions, whereas items 5 to 19 are rated on a 4-point Likert scale. Individual item scores yield seven components. A total score, ranging from 0 to 21, is obtained by adding together the seven component scores. A score higher than 5 suggests poor sleep quality.

#### Primary outcome measures

Insomnia severity will be assessed by the Insomnia Severity Index [21], which is a seven-item self-report questionnaire assessing the nature, severity, and impact of both nighttime and daytime symptoms of insomnia over the past week. It has been validated as a reliable and valid instrument that can be used to detect cases of insomnia in the population and is sensitive to treatment responses in clinical patients. A higher score represents greater insomnia severity. A minimally important treatment response can be indicated by a total score reduction > 7 points, while remission can be denoted by a total score of < 8 [21].

#### Secondary outcome measures

Interoception will be assessed by the Multidimensional Assessment of Interoceptive Awareness Scale [22]. The scale consists of 32 items that can capture changes in interoception associated with mind-body interventions. It comprises eight subscales, including noticing, not-distracting, not-worrying, attention regulation, emotional awareness, self-regulation, body listening, and trusting. A higher score indicates higher levels of interoceptive awareness.

The sleep-wake pattern will be assessed through wrist actigraphy. Wrist ActiGraph GT9X (ActiGraph, Pensacola, USA) will be used to measure sleep-wake patterns. Participants will be asked to put on an easy-to-wear device on the wrist of the non-dominant hand for 1 week at each measurement time period. Data will be collected continuously and stored in 1-min epochs. The sleep-wake pattern will be processed and analysed using ActiLife (V 6.11.9, Pensacola, USA).

#### Mediators

Health status will be assessed by the Chinese (Hong Kong) Short-Form-12 [23], which is a 12-item scale that can yield both physical health and mental health components. A higher respective summary score indicates better levels of health.

Rumination will be assessed by the rumination subscale from the Rumination-Reflection Questionnaire [24]. The subscale consists of 12 items that can assess recurrent thinking or rumination about the self, prompted by threats, losses, or injustices to the self. A higher score suggests greater ruminative tendencies.

Hyperarousal will be assessed by the hyperarousal scale [25]. It is a 26-item scale that can measure tendencies toward introspection, thinking about feelings, responding intensely to unexpected stimuli, and other behaviours that putatively involve cortical arousal. A higher score suggests a higher arousal level.

### Qualitative focus group interviews

One-fourth of the participants will be selected and invited from each intervention group for focus group interviews at T4. The format of the group interviews will be semi-structured, with a collaborative enquiry of the experiences gained from the respective interventions. The practicability of the programme, perceived therapeutic ingredients, and perceptions of the benefits regarding improvements in sleep disturbances from the participants’ perspectives will be explored and identified through the focus group interviews.

### Ethical considerations

This proposal has been approved by Human Subjects Ethics Sub-committee from the Hong Kong Polytechnic University (Reference number: HSEARS20210331002-01). An information sheet and a written consent form which has been formally approved by the ethics committee will be distributed to participants when they are invited to take part in the research project. Participation in this study will not make excessive discomfort or other injuries. Observation of mental state and any potential of self-harm behaviours will be closely taken throughout the processes of respective interventions or assessments. When necessary, participants would be advised to consult a professional. Individuals may experience some mild discomforts or tiredness during the MBTCC, MBI, or TCC programme. Such discomforts or fatigue, however, should be no greater than what they experience in everyday life. On the other hand, the participants may get direct therapeutic benefits of improving their sleep problems by taking part in respective interventions. Participants are free to decide whether to participate in this study, and they can revoke their consent and withdraw from the study at any time during the study, without any reason. This decision will not affect their medical care, nor will it cause any unpleasant results.

Informed consent will be obtained in accordance with the Declaration of Helsinki. The investigator will prepare the informed consent form which has been approved by the ethical committee. A properly executed, written, informed consent will be obtained from each participant prior to entering the participant into the trial. Information will be given in both oral and written form and participants will be given ample opportunity to inquire about details of the study. A copy of the signed consent form will be given to the participant, and the original will be maintained with the participant’s records. Any information obtained in this study will remain very strictly confidential, will be known to no one, and will be used for research purposes only. Codes, not names, are used on all test instruments to protect confidentiality. Data will be stored in encrypted files on the computer.

A participant may be discontinued from study treatment at any time if the participant or the investigator feels that it is not in the participant’s best interest to continue. There is a list of possible reasons for study treatment discontinuation: (1) the participant’s withdrawal of consent, (2) the participant is not compliant with study procedures, (3) the occurrence of adverse events so that the investigator would be in the best interest of the participant to discontinue his/her study treatment, (4) protocol violation requiring discontinuation of study treatment, and (5) lost to follow-up. All participants are free to withdraw from participation at any time, for any reasons, specified or unspecified, and without prejudice.

A trial steering committee which is composed of the first author, the corresponding author, and two invited independent expert members will be formed. The major goal of this committee is to monitor and supervise the progress of the study towards its overall objectives and ensure adherence to the protocol, patient safety, and consideration of new information relevant to the research question. In addition, the corresponding author will be responsible for all aspects of coordinating all local organizations including identifying potential recruits and taking consent. The committee will meet once every 3 months over the course of the trial to oversee conduct and progress.

### Data management

The investigator will prepare and maintain adequate and accurate source documents designed to record all observations and other pertinent data for each participant treated with different interventions. Participants will not be identified by name in the study database but will be identified by a participant code. The investigator is responsible for all information collected on participants enrolled in this study. All data collected during the course of this study must be reviewed and verified for completeness and accuracy by the investigator.

The data will be entered into a validated database. Database lock will occur once quality assurance procedures have been completed. All procedures for the handling and analysis of data will be conducted using good computing practices for the handling and analysis of data for clinical trials. After data have been entered into the study database, a system of computerized data validation checks will be implemented and applied to the database on a regular basis. The study database will be updated in accordance with the resolved queries. All changes to the study database will be documented.

The database is safeguarded against unauthorized access by established security procedures; appropriate backup copies of the database and related software files will be maintained. Databases are backed up by the database administrator in conjunction with any updates or changes to the database. At critical junctures of the protocol (e.g. production of interim reports and final reports), data for analysis is locked and cleaned per established procedures.

A data monitoring committee which is composed of the corresponding author, an independent biostatistician, and two invited independent expert members experienced in clinical trials will be formed. The major goal of this committee is to regularly review and maintain the confidentiality of the accumulating data in this trial. The independence of this committee is also to safeguard the interests of the participants and enhance the integrity of this trial. The committee will meet once every 3 months over the course of the trial to oversee conduct and progress.

### Data processing and analysis

The data will be analysed using SPSS version 26.0 on an intention-to-treat basis. To test the first study hypothesis, we will employ linear mixed models with restricted maximum likelihood estimation to test the individual growth model. This integrated random coefficient approach is usually conceptualized as a two-level model. Level 1, or the within-subject model, is the model used for repeated measures. Level 2, or the between-subjects model, is the model used for time-invariant variables between groups of individuals. The time factor, the grouping factor, and the interaction effect in mixed modelling will be examined. Additionally, the trend depicted in the least square mean plot will be evaluated to determine whether or not any trend-based differences exist between different groups. Hypothesis 1 would be supported if the models assessed and compared are fitted by using appropriate fit statistics. To test hypotheses 2 to 4, macro PROCESS SPSS and the bootstrapping approach will be used to assess the moderated mediation effects in the path model. Each mediator will be mean centred to form the interaction terms. The effects of covariates such as age or gender will be adjusted accordingly. The PROCESS macro is based on ordinary least squares regression. In addition, the mediating effect will be examined by data visualization techniques, such as Trellis coplot, also known as the conditional plot. Hypotheses 2 to 4 would be supported if any mediation effect exists.

The contents of the focus group interviews will be audiotaped and transcribed by a team of independent raters. Thematic analysis will be conducted with the help of the software NVivo (version 12) to store and manage the data. Coding and analysis will involve interpretative phenomenological analysis, which uses a bottom-up approach to determine the themes. Throughout the analysis, the codes will be identified from frequently used words or ideas in the transcripts. The codes will then be categorized into larger recurrent themes for further analysis. The research team will meet to discuss the significance of each theme and to triangulate the results with the quantitative findings.

## Discussion

MBIs and TCC are both perceived as mind-body interventions and share the common feature of a focus on breathing, but they may represent distinct approaches with different mechanisms and philosophical orientations. MBIs focus on stress management via changes in cognitive and behavioural responses and may therefore be conducive to treating rumination or psychological dysfunction. TCC, on the other hand, places a greater emphasis on physical health outcomes and the arousal-related physiological dimensions of sleep disturbance. Thus, *combining both MBIs and TCC has the potential to optimize improvements in sleep disturbance*. However, there is a dearth of evidence to guide clinical practice in regard to the best way to integrate these approaches. The current research study seeks to address this knowledge gap by investigating the combined effects of MBIs and TCC in the management of sleep disturbances.

This study has several potential limitations, including the risk of attrition and selection bias. In order to maintain the distinct benefit of randomization as a mechanism to prevent selection bias, an “as-randomized” analysis keeps participants in the group to which they were initially allocated. To avoid attrition bias, outcome data obtained from all participants are included in the data analysis, regardless of protocol adherence. That is, intention-to-treat analysis will be used as the ideal analysis strategy in this study. In addition, the study entails a long follow-up period. To achieve a high follow-up rate, we will ensure good communication between research personnel and participants, accessibility and convenience to nearby elderly community centres, effective communication channels, and appropriate incentives to continue and ensure that the study is of relevance to the participants.

Nevertheless, the present research project should determine the effects of an integrated MBTCC programme on sleep disturbances among Chinese older adults living in the community. The research findings can allow older adults or healthcare professionals to select appropriate practices. This will produce benefits, satisfaction, adherence, and sustainability in early interventions, which are potentially less costly and have fewer side effects. In addition, an evidence-based alternative intervention, as shown in this study, can help alleviate the burgeoning public health burden of sleep disturbances among older adults living in the community.

## Trial status

ClinicalTrials.gov ID: NCT05396092 (published on 24 May 2022)

The expected start date of recruitment: September 1, 2022

The expected end date of recruitment: April 18, 2025

## Data Availability

Any data required to support the protocol can be supplied on request. The datasets analysed during the current study and statistical code are available from the corresponding author on reasonable request, as is the full protocol.

## Abbreviations

CAM: Complementary and alternative medicine
MBIs: Mindfulness-based interventions
TCC: Tai Chi Chuan
MBTCC: Mindfulness-based Tai Chi Chuan
SHE: Sleep hygiene education
